# Pathophysiological links between traumatic brain injury and post-traumatic headaches

**DOI:** 10.12688/f1000research.9017.1

**Published:** 2016-08-31

**Authors:** Robert L. Ruff, Kayla Blake

**Affiliations:** 1 Case Western Reserve University, Cleveland, OH, USA; 2 Mayfield High School, Mayfield, OH, USA

**Keywords:** Traumatic brain injury, post traumatic headaches, post traumatic stress disorder, neuroinflammation

## Abstract

This article reviews possible ways that traumatic brain injury (TBI) can induce migraine-type post-traumatic headaches (PTHs) in children, adults, civilians, and military personnel. Several cerebral alterations resulting from TBI can foster the development of PTH, including neuroinflammation that can activate neural systems associated with migraine. TBI can also compromise the intrinsic pain modulation system and this would increase the level of perceived pain associated with PTH. Depression and anxiety disorders, especially post-traumatic stress disorder (PTSD), are associated with TBI and these psychological conditions can directly intensify PTH. Additionally, depression and PTSD alter sleep and this will increase headache severity and foster the genesis of PTH. This article also reviews the anatomic loci of injury associated with TBI and notes the overlap between areas of injury associated with TBI and PTSD.

## Introduction

The second International Classification of Headaches (ICHD-2) defines post-traumatic headaches (PTHs) as secondary headaches following traumatic brain injury (TBI) ^1^. No symptom-based criteria define PTH in the ICHD-2 criteria. PTH is more frequent after mild TBI (mTBI) compared with moderate or severe TBI ^2^ and can resemble one or more primary headache disorders ^3– 9^. The most common PTH patterns resemble two primary headache disorders—migraine or probable migraine and tension-type headaches—and migraine-type headaches are more prevalent ^3– 5, 8, 10, 11^. In one prospective study of adult civilian TBI associated with motor vehicle accidents, 58% of subjects continued to have PTH one year after their TBI ^12^. Migraine-like PTHs usually resemble migraine without aura ^5, 7, 8, 13, 14^. Neck injury in conjunction with TBI can result in cervicogenic headaches or pain that may resemble occipital neuralgia ^11, 15^. PTH can impair quality of life. Among veterans returning from Iraq or Afghanistan, the presence of PTH, especially if combined with one of the frequent TBI-associated psychological conditions of post-traumatic stress disorder (PTSD) or depression, diminishes the success of returning to work and other aspects of community reintegration ^16^.

Cervicogenic pain and tension-type PTH following TBI are often attributed to musculoskeletal injury caused by the trauma that produced TBI ^11, 15^. However, musculoskeletal trauma does not explain migraine-type PTH. This review describes possible mechanistic links between TBI and migraine to suggest how TBI can induce migraine-type PTH.

## Traumatic brain injury-induced neuroinflammation

Many brain tissue responses following TBI enhance the likelihood of, and perhaps directly cause, migraine-type PTH. For example, cellular injury increases the concentration of extracellular potassium, which will trigger neuronal depolarization and release of neurotransmitters that promote headaches ^17^. In addition, TBI triggers neuroinflammation, which enhances neuronal death and impairs recovery of function, but can also alter central nervous system (CNS) pain processing to induce migraine. Neuroinflammation is characterized by the activation of microglia; the release of pro-inflammatory chemicals, including chemokines, specific interleukins, and tumor necrosis factor-alpha (TNF-α); and possibly the invasion of the CNS by inflammatory lymphocytes and phagocytic white cells coming through cerebral blood vessels. Neuroinflammation is a central element in the development of chronic traumatic encephalopathy (CTE) associated with repeated sports-associated TBI events ^18– 22^ and includes headache as part of the symptom spectrum ^18^. Non-hemorrhagic, closed-head TBI activates microglia, leading to the production of inflammatory and pro-inflammatory molecules ^20, 23– 28^, and even minor experimental TBI induces neuroinflammation by activating microglia ^29^. Moderate and severe TBI can disrupt the blood-brain barrier, allowing invasion of neutrophils from leaky blood vessels ^23, 24, 28^. Neuroinflammation develops quickly after TBI as shown by a post-mortem study of civilians in which the brains analyzed after death associated with acute TBI demonstrated elevated mRNA levels of interleukin-1 beta (IL-1β), IL-6, and IL-8 and TNF-α within minutes of TBI ^30^.

Neuroinflammation can be modulated in several ways. Microglial activation is regulated by several factors, including poly (ADP-ribose) polymerase-1 (PARP-1) and the metabotropic glutamate receptor 5 (mGluR5). PARP-1 is present in neurons and glia. It is the most abundant member of a family of enzymes that attach ADP-ribose polymers onto proteins. PARP-1 resides in the nucleus and is activated by DNA strand breaks ^31, 32^. At low levels of activation, PARP-1 facilitates DNA base excision repair by poly-ADP-ribosylation of proteins involved in DNA repair. PARP-1 recruits DNA repair proteins to DNA break sites to restore DNA integrity. However, PARP-1 activation can damage neurons and glia after TBI ^33, 34^. PARP-1-mediated neuronal cell death may be partially due to PARP-1 activation depleting NAD ^+^ and ATP ^31^. Additionally, PARP-1 may initiate neuronal death through the release of apoptosis-inducing factor and impairing mitochondrial function and glycolytic metabolism ^32, 35, 36^. PARP-1-dependent nuclear factor-kappa-B activation induces the expression of pro-inflammatory genes and activates microglia ^37, 38^. Activated microglia release multiple types of neurotoxic molecules. Therefore, strong activation of PARP-1 due to TBI may convert PARP-1 from being an agent of DNA repair to a trigger of neuronal injury and neuroinflammation. In support of PARP-1 contributing to CNS damage in TBI, PARP-1 inhibition reduces microglial activation and functional impairment and increases neuron survival in experimental models of TBI ^38– 40^. The activation of mGluR5 decreases microglial activation and reduces microglial release of pro-inflammatory factors, which reduces neuronal loss and improves functional recovery after experimental TBI ^41^.

Peroxisome proliferator-activated receptors (PPARs) are ligand-activated transcription factors that are members of the super family of nuclear hormone receptors ^42, 43^. PPARs control many physiological functions, including glucose absorption, lipid balance, and cell growth and differentiation. Among the several PPAR isoforms, the activation of PPAR-α and PPAR-γ exerts neuroprotective actions in acute cerebral injury such as TBI ^44– 47^ and stroke ^48^ and reduces neuronal injury in animal models of cerebral degenerative disorders, including Parkinson’s disease ^49– 52^, amyotrophic lateral sclerosis ^53^, and Alzheimer’s disease ^54^. Treatment with a PPAR agonist after experimental TBI prevents microglial activation, reduces cognitive impairment, and reduces neuron death ^46^.

The role of neuroinflammation in brain injury and recovery following TBI is complex. IL-6 is an inflammatory mediator. IL-6 is one of the first cytokines produced after TBI. Chemically suppressing IL-6, IL-1β, and TNF-α in mice reduces brain edema and apoptosis ^55^. However, selective knockout of IL-6 in mice impaired functional recovery and enhanced the production of IL-1β following TBI compared with controls ^56^. In children, IL-6 and nerve growth factor upregulation correlated with improved outcome after severe TBI ^57^. Activation of adenosine 2A receptors can have pro-inflammatory or anti-inflammatory actions in the brain depending on the cell types containing the receptor and the degree of receptor activation ^58^. Chemokines may have a complex role in brain response to TBI. Deletion of the chemokine ligand 2 receptor (CCR2) resulted in reduced monocyte infiltration of the cortex along with diminished neuronal death and axon injury in an animal model of TBI ^20^. However, CCR2 deletion did not change microglia activation. Additionally, CCR2 deletion was associated with increased tau-protein mislocalization to the neuron cell bodies in the cortex and increased levels of phosphorylated tau in the hippocampus ^20^. The changes in tau associated with CCR2 deletion resemble findings described in CTE ^19, 21^.

Physical exercise before experimental TBI reduces neuroinflammation and improves functional recovery following fluid-percussion TBI in rats ^59^. The protective effect of physical conditioning prior to TBI might influence recovery after TBI in athletes and active military personnel. The mechanisms of exercise reducing neuroinflammation are under investigation.

## Possible links between neuroinflammation and migraine

Neuroinflammation alters the functions and sensitivities of meningeal and cerebrovascular structures ^60^ that are involved in the development and progression of head pain and other features of migraine ^61^. Additionally, inflammation and other responses to injury can enhance neuronal excitability ^62– 64^, which could potentiate the genesis of cortical spreading depression (CSD). CSD is an important element in migraine, particularly migraine aura ^61^. Thus, altering the properties of cerebral, meningeal, and cerebrovascular structures involved in migraine could be a link between TBI and PTH.

Neuroinflammation may play a role in brain recovery after TBI; however, exaggerated inflammation may lead to a state of hyperexcitability involving structures that could potentiate the development of migraine and other headaches. The headache phase of migraine is associated with the release of vasoactive neuropeptides by the trigeminovascular system, vasodilation of the extracerebral intracranial arteries, and increased nociceptive transmission within the central trigeminocervical complex ^65^. Cady proposed that migraine symptoms evolve as the pathology of inflammation and neural sensitization progress ^66^. Tension headache symptoms appeared early in the course of inflammation. As inflammation and neural hyperexcitability advance, the pattern of headaches shifted from tension-like to migraneous headaches to classic migraine and other forms of migraine. This convergence model of headaches provides a conceptual framework to consider how PTH can result from post-TBI neuroinflammation. Several reviews addressed the connections between neuroinflammation and migraine ^67– 69^.

Hyperexcitability of primary trigeminal afferent nociceptive neurons can initiate or aggravate neuroinflammation involving meningeal blood vessels. Hyperexcitability resulting from inflammation of trigeminal nerve branches mediates the throbbing head pain of migraine ^61^. The throbbing character of migraine may reflect pain signals carried by afferent pain fibers that innervate pulsating meningeal blood vessels. CSD is involved in the genesis of migraine aura and activation of meningeal pain receptors ^69^. CSD may sensitize and activate meningeal nociceptors through the release of vasoactive pain-stimulating chemicals such as substance P, leading to the activation of mast cells and macrophages, which in turn release inflammatory mediators, including cytokines ^69^. Another factor involved in migraine is the neuropeptide calcitonin gene-related peptide (CGRP) ^70^. CGRP modulates pain by peripheral and central mechanisms. Outside the brain, meningeal nociceptors are activated by CSD triggering the release of CGRP, which induces vasodilation and edema ^68^. CGRP can also sensitize meningeal nociceptors producing positive feedback that increases the activity of meningeal nociceptors. Additionally, glia in the trigeminal ganglion release a CGRP-like peptide called procalcitonin, which also induces neuroinflammation ^68, 70^. The transient receptor potential V1 channel (TRPV1) potentiates the release of CGRP in nociceptive trigeminal ganglia neurons and TBI activates TRPV1 ^71^.

## Traumatic brain injury can impair pain modulation

Pain intensity is based in part on the activity of intrinsic pain modulation systems that diminish pain transmission and alter the perceived intensity of painful stimuli ^72^. Pain modulation is impaired in people with chronic PTH ^73^. Diminished ability to reduce the perceived intensity of pain could prolong the duration and increase the intensity of PTH ^73^. Impaired pain modulation in people with PTH results from disruption of the intrinsic pain modulation system and psychological changes that can alter pain perception. We will first consider TBI-induced alterations in the intrinsic pain modulation system.

Serotinergic (5-HT) neurons are important in pain modulation ^72^, and migraine is associated with 5-HT pathways. The triptan class of migraine abortive medications are agonists of 5-HT(1B/1D) receptors ^74^. TBI could facilitate PTH by reducing the activity of 5-HT neurons that counter migraine. Activating 5-HT receptors inhibits the release of CGRP and other vasoactive neuropeptides from trigeminal nerves ^68, 70^. 5-HT(1B/1D) receptors constrict painfully dilated cerebral blood vessels and inhibit nociceptive neurotransmission in trigeminal pathways ^65^. Trigeminal ganglion neurons project centrally to several locations, including pain pathways and the nucleus tractus solitarius (NTS), which is involved in the sensation of nausea ^75^.

TBI targets 5-HT pathways in the brain. Long serotonergic axons may be damaged by diffuse axonal injury (DAI) associated with TBI. TBI causes DAI because long axons are stretched by the physical distortion of the brain which occurs with all forms of diffuse TBI ^76, 77^. An imaging study of people with chronic PTH demonstrated central white matter damage that correlated with the presence of chronic PTH ^78^. The imaging study could not identify whether the white matter damage involved components of the pain modulation system; however, the locations of white matter injury associated with chronic PTH included regions containing pain modulation fibers that interface with the amygdalae and other cerebral nuclei ^72^.

Serotonergic neuron cell bodies are organized into two distinct groups: caudal (medulla and caudal pons) and rostral (midbrain and rostral pons) ^79^. Additionally, there are serotonergic neuron cell bodies in the medullary reticular formation (involved in alertness) and other non-raphe regions such as the hippocampus and the substantia nigra ^79^. There is a biphasic change in the levels of 5-HT in cerebral extracellular and perivascular spaces after TBI. For the first few days after TBI, 5-HT levels are elevated ^80– 82^. Elevation of 5-HT levels may result from stretch-induced firing of serotonergic nerve fibers and release of 5-HT from inflammatory cells. The acute elevation of 5-HT levels is associated with breakdown of the blood-brain barrier and cerebral edema ^83^. Levels of 5-HT and its metabolites decline several days after brain injury and can remain persistently reduced ^84, 85^. Persistent damage to 5-HT pathways, particularly serotonergic innervation of cerebral blood vessels, may induce migraine following TBI by disrupting normal serotonergic suppression of patterns of neuronal and cerebrovascular activity associated with migraine ^82^. Additionally, trigeminovascular afferent activation of NTS cells can induce symptoms such as nausea. Nausea can be suppressed by activating 5-HT(1B/1D) receptors that inhibit NTS cells associated with migraine symptoms such as nausea ^75^. Thus, somatic symptoms associated with migraine such as nausea may be potentiated by over-activity of injured trigeminal neurons and reduction in serotonergic tone following TBI.

## Traumatic brain injury-associated psychological changes that may potentiate post-traumatic headache

Psychological states of depression and anxiety can increase the perceived intensity of pain such as PTH ^72^. Among civilians with migraine, the frequency and severity of migraine attacks are associated with PTSD ^86^. The presence of mTBI may increase the likelihood that a psychologically traumatic event results in PTSD ^87^. In rats, explosions that produce mTBI induce PTSD-like behavior ^88^. Among combat veterans with mTBI-associated explosions, there is a dose-response relationship between the number of TBI events and the likelihood of an individual developing PTSD ^4, 89, 90^. A study of anxiety disorders in children following TBI found that 8.5% of children developed anxiety disorders, usually PTSD, within 6 months of the TBI and those with mTBI had the greatest likelihood of developing PTSD ^91^. A national study of civilian trauma in Australia reported that the prevalence of new-onset depression was 9% after trauma. The presence of TBI with the trauma did not seem to influence the development of depression. PTSD prevalence following mTBI was 6%, which was about 1.9-fold higher than following trauma without TBI ^92^. PTSD and mTBI are associated in combat personnel. About 40% of military personnel and veterans with combat-acquired mTBI had PTSD ^11, 93^. The incidence rates of PTSD were 44% for soldiers recently deployed in Iraq who experienced an episode of loss of consciousness due to mTBI, 16% for soldiers with other injuries, and 9% for uninjured soldiers ^93^.

Depression and PTSD are frequently present in civilians with chronic PTH ^94– 97^. A study of psychological issues in civilians with chronic PTH found that 30% of subjects with PTH met criteria for PTSD and depression ^98^. Although experiencing chronic pain can alter an individual’s psychological state, data suggest that PTSD is probably linked to the traumatic event rather than to chronicity of pain ^99^. A meta-analysis of studies of civilian and military TBI concluded that PTSD may modulate the intensity and severity of chronic PTH but that TBI independently correlated with the genesis of chronic PTH ^2, 100^. A longitudinal study of Iraq and Afghanistan war veterans initially treated by the Department of Veterans Affairs in 2008 and followed through 2011 reported that PTH correlated with mTBI ^95^. Additionally, coexistence of PTSD, depression, or both increased the likelihood of chronic PTH beyond that associated with mTBI alone ^95^.

PTSD and depression can intensify PTH by disrupting sleep and this reduces the threshold for pain. PTSD disrupts sleep, and the impaired sleep associated with PTSD intensifies pain symptoms ^101– 104^. Improving the sleep of US combat veterans who sustained mTBI in Iraq or Afghanistan reduced PTSD severity and decreased the frequency and intensity of PTH episodes ^102, 103^. Depression is associated with impaired sleep, and impaired sleep can exacerbate depression ^105^. Sleep deprivation and depression independently lower the pain threshold ^106^. Several studies found that sleep deprivation lowers pain thresholds ^107– 109^.

PTSD is associated with chronic pain disorders, and PTSD has complex interactions with pain thresholds ^110^. One report indicated that PTSD increases the pain threshold but that supra-threshold pain was perceived as excessively intense ^111^. PTSD disrupts sleep by inducing nightmares ^4, 101– 103, 112– 116^. Prazosin, an alpha-1 adrenergic receptor antagonist that passes through the blood-brain barrier, reduces PTSD-associated nightmares ^102, 103, 112– 117^, improves cognitive performance, and reduces PTH frequency and severity ^4, 102, 103^.

## Possible relationships between mild traumatic brain injury and post-traumatic stress disorder

We suggest that cerebral injury associated with mTBI predisposes individuals to develop PTSD in response to psychologically traumatic events associated with the mTBI. A hypothesis on the neural basis of PTSD is that PTSD results from over-activation of the amygdalae due to loss of inhibitory regulation by the ventromedial prefrontal cortex and hippocampus ^118^. Pitman *et al*. ^119^ and Sherin and Nemeroff ^120^ discuss the roles of hippocampus and ventromedial prefrontal cortex regulation of the amygdalae in the genesis of PTSD. Brain areas with significantly altered levels of activity identified by functional imaging in PTSD include ventromedial prefrontal and medial temporal lobes ^121– 123^ which are damaged in mTBI ^124– 137^. Single-photon emission tomography demonstrated reduced benzodiazepine receptor binding in the prefrontal cortex of people with PTSD ^138^. Prefrontal cortex activity in response to emotional stimuli is altered in PTSD ^139^. In combat-associated TBI with PTSD, PTSD severity was associated with the presence of magnetic resonance imaging lesions involving the prefrontal cortex ^140^.

In addition to imaging evidence that the ventromedial prefrontal cortex is altered in PTSD, the neurological examination finding of impaired olfaction supports altered function of the ventromedial prefrontal cortex in people with PTSD associated with mTBI ^3, 4, 89, 92^. The initial site of cortical processing of olfactory information is in the ventromedial prefrontal cortex ^141^. Impaired olfaction was the most frequently recognized chronic/persistent neurological deficit on clinical physical examination ^4^. TBI compromises olfaction by shearing the olfactory nerves traversing the cribriform plate or ventromedial prefrontal cortex injury or both ^141^. Injured olfactory nerve fibers can recover over time, leading to improvement of olfaction in the first year after TBI ^142^. Veterans in the study of olfaction after mTBI were evaluated more than one year after their last TBI; thus, the reported persistent reduction in olfaction likely resulted from damage to the ventromedial prefrontal cortex ^4^.

A study of US military personnel who served in Iraq or Afghanistan and sustained combat TBI, predominantly mTBI, reported that 29% of subjects had white matter lesions in at least two areas of interest ^127^. The areas of injury were ventromedial prefrontal cortex, cingulum, and middle cerebellar peduncles. The finding of ventromedial prefrontal cortex injury associated with combat mTBI provides a structural explanation for impaired olfaction in combat mTBI ^4, 92^. Further support for a structural basis for both impaired olfaction and PTSD with combat mTBI is that both impaired olfaction and the presence and severity of PTSD correlated with the number of mTBI events ^4, 92^.

Animal studies suggest that the neural elements associated with PTSD, including the amygdalae, ventromedial prefrontal cortex, and hippocampus, have unique responses to psychological stress that may perpetuate and enhance the severity of PTSD. Placing rats in prolonged stressful environments enhanced dendritic arborization of neurons in the amygdalae ^143, 144^ and produced dendrite atrophy in the hippocampus ^143^. Consequently, continued stress, as might be produced by difficulty functioning within society because of chronic PTH, could lead to structural brain changes that perpetuate and intensify PTSD. For combat veterans, the frequency and severity of PTH are associated with PTSD severity ^4^. Consequently, PTH and PTSD could augment each other via cerebral structural changes.

Studies of Vietnam-era veterans show that subtle neurological deficits can increase the risk of developing PTSD ^145^. In studies of monozygotic twins in which one twin was in combat and the other was not, among twin pairs where one had combat-associated PTSD, both twins had a higher prevalence of subtle neurological deficits ^145^ and gray matter abnormalities in the right hippocampus, pregenual anterior cingulate cortex, and left and right insular cortex ^146^. The twin studies showed that subtle, genetically based neurological deficits can potentiate the genesis of PTSD.

The twin studies contrast with the Vietnam Head Injury Program (VHIP), in which veterans with combat penetrating severe TBIs involving the right amygdala or bilateral ventromedial prefrontal cortex had reduced prevalence of PTSD ^119^. One explanation for the differences between the VHIP and twin studies is that the subjects in the VHIP study had severe TBI compared with the subtle deficits of participants in the twin studies. Mild cerebral injury could foster PTSD genesis by impairing normal interactions among the amygdala, ventromedial prefrontal cortex, and hippocampus, whereas the more severe injuries in the VHIP study may prevent “the ‘super-normal’ levels of fear/anxiety that define PTSD” ^119^. Thus, mild injury to specific areas of the brain may potentiate the genesis of PTSD, whereas obliteration of these areas may prevent PTSD from developing. In childhood TBI, PTSD and other anxiety disorders correlated with mild injury to the superior frontal gyrus, anterior frontal white matter, and ventromedial prefrontal gray matter ^93, 123^. Overall, the risk of developing PTSD following combat TBI is greater for mTBI compared with severe TBI ^147^. Children with mTBI are more likely to develop PTSD than children with severe TBI ^93^.

In summary, TBI can induce PTH by several mechanisms. TBI can induce neuroinflammation that activates structures associated with headache genesis, especially migraine. CNS damage resulting from TBI will impair pain modulation and enhance headache genesis. The CNS injury resulting from TBI can potentiate the development of depression and anxiety disorders, including PTSD that can intensify PTH. The areas of the brain injured in TBI overlap with the areas of impaired function associated with PTSD. The complex nature of PTH suggests that their treatment should include interventions that address psychological issues such as depression and PTSD and impaired sleep and that directly address pain symptoms associated with PTH.

Many questions remain regarding the genesis of PTH. We believe that an important issue to examine is whether some individuals are more susceptible to developing persisting PTH. Psychological conditions and secondary gain can influence the likelihood that an individual will develop PTH ^148^. Psychological resilience enhances the ability of individuals to recover from psychological trauma and appears to reduce the likelihood that an individual will recover from TBI with prolonged post-concussion symptoms, including PTH ^149^. The twin studies demonstrated that subtle, genetically based neurological deficits influence the genesis of PTSD ^145, 146^. We suggest that genetically based or acquired alterations in brain structure may also influence the likelihood that an individual will develop PTH.
